# Intraprocedure Artificial Intelligence Alert System for Colonoscopy Examination

**DOI:** 10.3390/s23031211

**Published:** 2023-01-20

**Authors:** Chen-Ming Hsu, Chien-Chang Hsu, Zhe-Ming Hsu, Tsung-Hsing Chen, Tony Kuo

**Affiliations:** 1 Department of Gastroenterology and Hepatology, Taoyuan Chang Gung Memorial Hospital, Taoyuan 333, Taiwan; 2 Department of Gastroenterology and Hepatology, Linkou Chang Gung Memorial Hospital, Taoyuan 333, Taiwan; 3 College of Medicine, Chang Gung University, Taoyuan 333, Taiwan; 4 Department of Computer Science and Information Engineering, Fu-Jen Catholic University, New Taipei 242, Taiwan

**Keywords:** colonoscopy, intra-procedure alert system, dynamic colon polyp image, blurred image detection, foreign body detection, polyp detection, deep learning

## Abstract

Colonoscopy is a valuable tool for preventing and reducing the incidence and mortality of colorectal cancer. Although several computer-aided colorectal polyp detection and diagnosis systems have been proposed for clinical application, many remain susceptible to interference problems, including low image clarity, unevenness, and low accuracy for the analysis of dynamic images; these drawbacks affect the robustness and practicality of these systems. This study proposed an intraprocedure alert system for colonoscopy examination developed on the basis of deep learning. The proposed system features blurred image detection, foreign body detection, and polyp detection modules facilitated by convolutional neural networks. The training and validation datasets included high-quality images and low-quality images, including blurred images and those containing folds, fecal matter, and opaque water. For the detection of blurred images and images containing folds, fecal matter, and opaque water, the accuracy rate was 96.2%. Furthermore, the study results indicated a per-polyp detection accuracy of 100% when the system was applied to video images. The recall rates for high-quality image frames and polyp image frames were 95.7% and 92%, respectively. The overall alert accuracy rate and the false-positive rate of low quality for video images obtained through per-frame analysis were 95.3% and 0.18%, respectively. The proposed system can be used to alert colonoscopists to the need to slow their procedural speed or to perform flush or lumen inflation in cases where the colonoscope is being moved too rapidly, where fecal residue is present in the intestinal tract, or where the colon has been inadequately distended.

## 1. Introduction

Colonoscopy is a valuable tool for detecting colorectal diseases. Chromoendoscopy is often used in the diagnosis of polyps through the enhancement of the color, vascular structure, and surface morphology of polyp lesions [1]. Generally, chromoendoscopy is divided into dye- and equipment-based types [2]. Equipment-based chromoendoscopy, or virtual chromoendoscopy, is the type that is most extensively used in current clinical practice; it involves narrowband imaging, flexible spectral imaging color enhancement, i-scan, and blue laser imaging [3]. When used in conjunction with magnifying colonoscopy, such equipment-based techniques can help to accurately distinguish non-neoplastic and neoplastic lesions, help predict the depth of invasion, and assist endoscopists in using the correct treatment methods [4], thereby effectively reducing the incidence and mortality of colorectal cancer [5].

Patients undergoing colonoscopy are required to maintain a low-residue diet and take laxatives to empty their bowels of excrement to allow the colonoscopists to have a clear view of the intestinal mucosa. While performing a colonoscopy, a colonoscopist inserts a colonoscope into the patient’s anus and further guides it into their bowel lumen, passing through the rectum, sigmoid colon, descending colon, transverse colon, and ascending colon before finally reaching the cecum. The colonoscopist then slowly pulls back the colonoscope from the cecum and may push it forward or pull it farther back as necessary to examine the mucosa in detail. Polypectomy or biopsy may be performed if a polyp or lesion is detected. Among the quality indicators of colonoscopy [6], the adenoma detection rate (ADR), quality of bowel cleansing, withdrawal time, cecal intubation rate, and complete polypectomy rate are closely correlated with the occurrence of colorectal cancer after colonoscopy [7]. However, considerable variance in ADRs may occur among endoscopists, which could, in turn, diminish the clinical benefits of colonoscopy.

Several deep learning–based systems have been formulated for the diagnosis and detection of colorectal polyps [8,9,10,11,12,13,14]. For example, Chen et al. [15] used deep neural networks to distinguish narrowband images of neoplastic and hyperplastic polyps at the Tri-Service General Hospital in Taiwan. Park et al. [16] used a convolutional neural network (CNN) to classify polyps captured in colonoscopic images. Shin et al. [17] proposed a method based on a region-based CNN (R-CNN) that engages in false-positive and offline learning for polyp detection. Ren et al. [18] used an R-CNN to segment polyps in images. Wang et al. [19] used a segmentation network called SegNet to screen for polyps. Zheng et al. [20] employed a you only look once (YOLO) model to detect polyps in white-light and narrowband images. Hsu et al. [3] used grayscale images and a CNN-based deep network to enhance features in colonoscopic images, detect the location of colorectal polyps, and identify polyp types. Nogueira-Rodríguez et al. [21] used an updated version of the YOLO model, called YOLOv3, for real-time polyp detection. Li et al. [22] collected endoscopic images, publicly available on the Internet, including those in the MICCAI 2017, CVC colon DB, GLRC, and KUMC datasets; the researchers extracted the polyp images of interest and employed multiple deep learning models—including Faster R-CNN, YOLOv3, RetinaNet, DetNet, RefineDet, YOLOv4, and adaptive training sample selection—for polyp detection and identification. However, many of the systems proposed in these studies rely on perfect manually captured images or require magnified images for model training, verification, and testing. Thus, the result may be a model that is vulnerable, has low accuracy, or yields excessive false positives, which would be difficult to apply in clinical settings [23].

**Figure 1:**
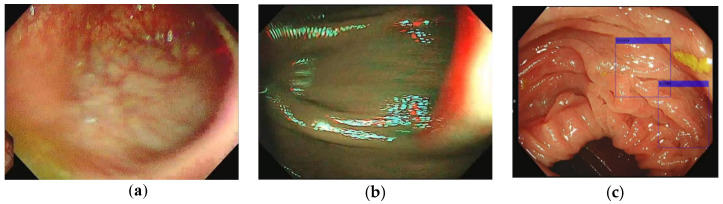
Poor colonoscopic image quality types. (**a**) Out-of-focus image, (**b**) Camera shake, (**c**) Insufficient air insufflation resulting false positive.

The clarity of images of the colorectal mucosa strongly affects the quality of colonoscopy. The factors behind low colonoscopic image quality include inadequate bowel preparation, insufficient air or carbon dioxide insufflation, lens fogging or a colonoscopic lens stained with fecal matter, and blurred images (Figure 1). In addition, a patient not complying with the instruction to consume a low-residue diet or not taking their prescribed dose of bowel-cleansing medication can result in the presence of residual fecal material or water in their colorectal lumen, and insufficient air or carbon dioxide insufflation may lead to poor inflation of the lumen, which may hinder the clear identification of abnormalities. Finally, lens fogging or a colonoscopic lens stained by fecal matter or tissue fluid can hinder image recognition. 

Blurred images refer to images rendered out of focus because of motion resulting from the rapid withdrawal of the colonoscope or from withdrawal with an unsteady hand, either of which can prevent the accurate observation and identification of lesions. Colonoscopists may miss lesions because of low image quality, and machine vision systems, whose accuracy is particularly dependent on image quality, may perform much worse if the problem of poor image quality is not addressed. Hassan et al. [24] reported an average rate of 3.2 false positives when a computer-aided polyp detection system was used in colonoscopy. Most of the false positives were due to bowel content, artifacts from the mucosal wall, air bubbles, fecal water, or blood in the intestinal tract. Another factor behind these false-positive cases was computer-aided diagnoses made on the basis of images obtained under insufficient air insufflation. Rutter et al. [25] suggested that patients may still develop colorectal cancer, despite having undergone screening; they reported that 2.5–7.7% of patients had colorectal cancers within 3–5 years after receiving a colonoscopy, primarily due to inaccurate screening results.

Sharp and clear images are necessary for the computer-aided detection and identification of colorectal polyps and lesions. The technique of searching for and identifying the shape, texture, and morphology of protrusions in the intestinal tract is akin to the identification of foreign object damage (FOD) on an airport runway, which severely hinders flight and passenger safety [26,27]. Some FOD detection systems used in airports worldwide are based on optical detection with deep learning or hybrid techniques. Thus, it is crucial to establish a robust intraprocedure artificial intelligence (AI) alert system to reduce the burden of clinicians involved in colonoscopy.

This study proposed an alert system for colonoscopic examinations that uses deep learning to alert the colonoscopist with a message when the image is blurred because of rapid movement or when fecal matter or water in the lumen, insufficient air inflation, or polyps are detected. Colonoscopists should pay attention to any abnormalities during examination to avoid missing lesions and to improve the quality of colonoscopy. A CNN model was used to determine when an alert message ought to be sent. The experimental results revealed that the number of polyps of each case identified by the proposed system is the same as the number of polyps detected by endoscopists through per-polyp analysis. In addition, the sensitivity rates for the detection of high-quality images and the detection of polyps were 92% and 95.74%, respectively, and the false alarm rate of low-quality images was 0.18%. The proposed system can be employed by clinicians to improve the quality of colonoscopies.

The remainder of this paper is organized as follows: Section 2 describes the materials and methods; Section 3 details the evaluation experiments; and Section 4 discusses the results and concludes the paper.

## 2. Materials and Methods

The training and validation datasets used for the proposed system were obtained from the PolypsSet dataset [22] and Chang Gung Memorial Hospital (Table 1). In total, 3750 low-quality images and 2500 high-quality images were selected by experienced colonoscopists from the experimental datasets. The low-quality images included blurred images and images that contained folds, fecal matter, and opaque water. High-quality images were defined as images with clear and well-distended colon lumen and with no fecal residue or opaque fluid. Each image had a resolution of 640 × 480 pixels and was a TIF file. Among the low-quality images, the number of blurred images and the number of images containing folds, fecal matter, and opaque fluid were 2500 and 1250, respectively. In addition, the number of high-quality images containing polyps and the number of those containing no polyps were 1250 and 1250, respectively. The test dataset was derived from six videos (Table 2) that had been obtained for this study from Linkou Chang Gung Memorial Hospital. Each video was in MKV format, lasted approximately 15 min, and displayed 30 frames per second. The colonoscope model was CF-H290L/I, which featured a 170° angle of view, a forward-viewing direction of view, and a depth of field of 5–100 mm. After the images were de-identified and all of the non-intestinal information was cropped from the images, the images had a resolution of 720 × 960 pixels. After the deletion of the first 3–10 min portion of each video, which showed the insertion of the colonoscope into the cecum, the remaining footage was employed for polyp detection and identification at 3 frames per second. Among the dynamic images obtained from the videos, the number of blurred images and the number of images containing folds, fecal matter, and opaque water were 8716 and 1967, respectively, and the numbers of high-quality normal images and polyp images were 399 and 50, respectively. All of the videos featured one polyp, except for Case #1, which featured two polyps (Table 3). Polyp detection was performed using a CNN model for classification, and the training dataset comprised 612 images from the CVC-ClinicDB dataset and 500 images from the PolypsSet dataset (Table 4).

**Figure 2:**
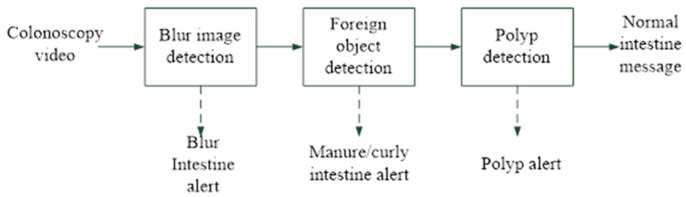
System architecture.

**Figure 3:**
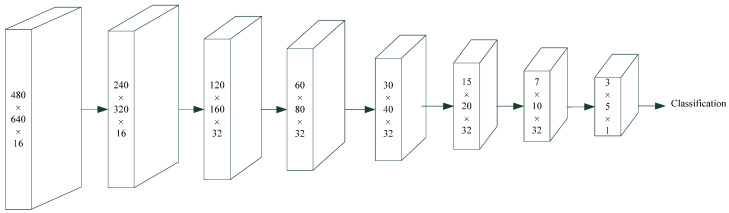
Alert system architecture.

**Figure 4:**
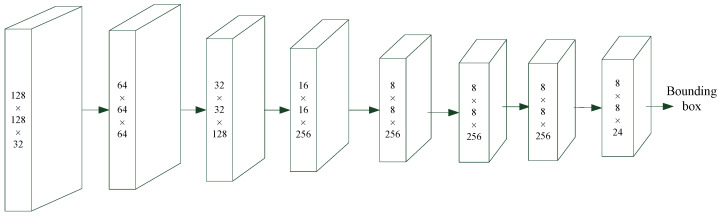
Polyp detection architecture.

Figure 2 shows the architecture of the proposed intraprocedure alert system, which provides blurred image detection, foreign body detection, and polyp detection. Blurred image detection is used to identify blurred images that have occurred due to camera shaking, to the colonoscope being withdrawn too rapidly, or to the lens being stained with fecal matter or opaque fluid. The presence of a colon fold and fecal matter or methods for fluid detection are used to indicate abnormal protrusions that may be haustral folds and creases or fecal residue. Finally, polyp detection is used to identify polyp protrusions in the colon lumen [3]. All of these functions are provided by the proposed CNN deep learning model. Figure 3 and Table 5 present the proposed CNN deep learning model architecture for the detection of blurred images, fecal matter, opaque water, and colon folds. Figure 4 and Table 6 present the polyp detection architecture for feature extraction and the bounding box transformation layer for the result output. Notably, polyp detection was performed on images from the six videos to verify the effectiveness of the system in identifying false alerts after low-quality images were excluded.

The size of each input image was measured in terms of the width (W) × Height (H) × filter number (N). All of the images were adjusted to fit the specification of the CNN deep learning model. We employed convolution, batch normalization, a rectified linear unit, and maximum pooling operations to conduct feature extraction [3]. Table 5 and Table 6 show the filters, size/stride, and output image size of each operation. A classification conversion layer was used to distinguish blurred images from non-polyp foreign body images. In addition, fully connected, softmax, and classification output layers were used for classification.

## 3. Experimental Results

**Figure 5:**
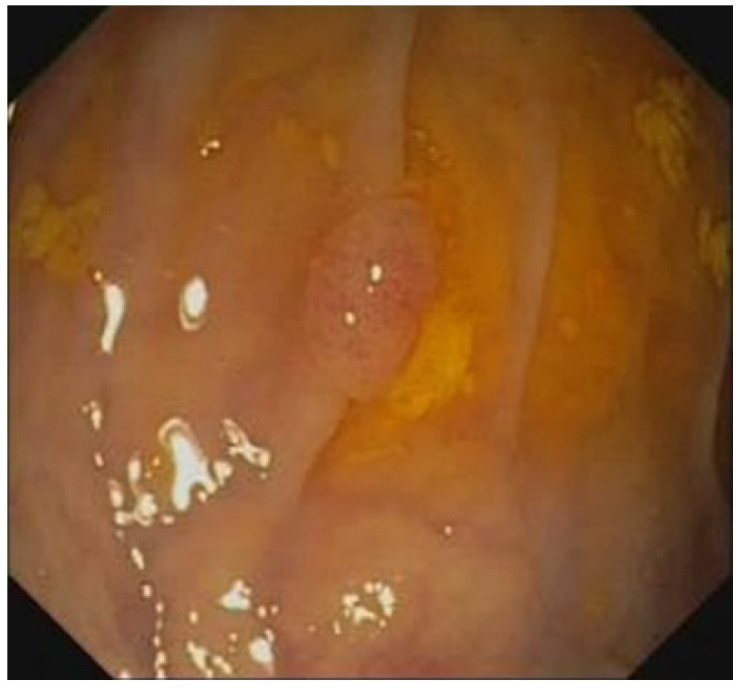
Polyp erroneously signaled as fecal matter or water.

**Figure 6:**
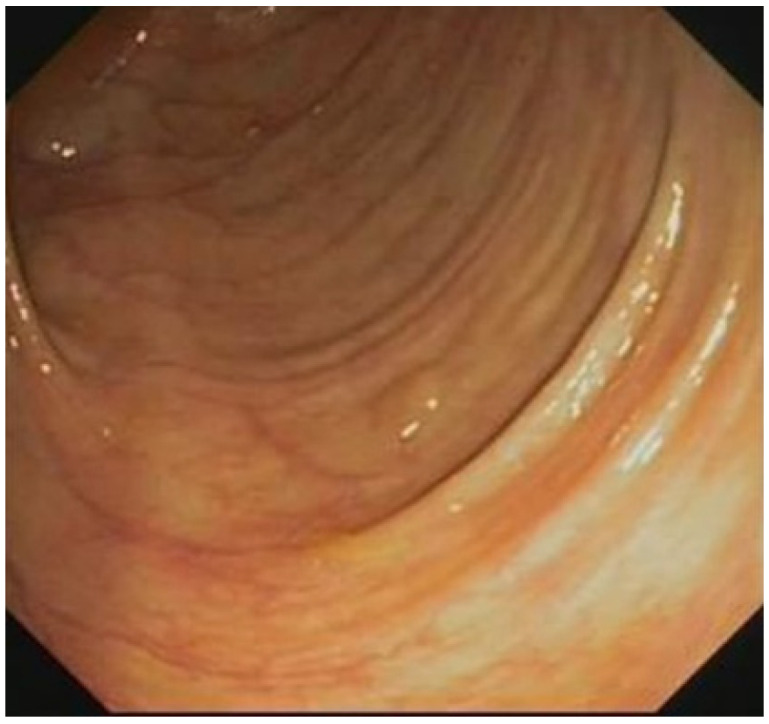
Polyp erroneously signaled as a colon fold.

Table 7 and Table 8 present the training and validation datasets for the low-quality images, respectively, including blurred images and foreign body images, which numbered 5000 and 2500, respectively. Table 9 and Table 10 present the respective confusion matrices for the blurred image and foreign body detection. “TP,” “FN,” “TN,” and “FP” denote “true positive,” “false negative,” “true negative,” and “false positive,” respectively. A five-fold cross-validation method was used to verify the performance effectiveness. Table 11 presents the calculation method for the classification performance index, and Table 12 presents the classification performance for the blurred image and foreign body detection. For the detection of blurred images, the accuracy, precision, recall, F1-measure, and F2-measure were 96.2%, 98.8%, 93.6%, 96.1%, and 94.6%, respectively. For the detection of folds, fecal matter, and opaque water images, the accuracy, precision, recall, F1-measure, and F2-measure were 96.2%, 97.5%, 94.8%, 96.1%, and 95.3%, respectively. Among the factors behind the occurrence of false positives was an increased concentration of fecal matter or water near a polyp or the presence of a crease next to a tiny polyp, which tended to cause the system to issue an alert for the presence of fecal matter or colon folds (Figure 5 and Figure 6).

Table 13 and Table 14 present the actual numbers of polyps detected after blurred images and images containing fecal matter or water and colon folds were excluded, respectively, along with the identification accuracy levels for the high-quality normal colon images and polyp images. The numbers of polyps, high-quality normal image frames, and polyp image frames were 7, 399, and 50, respectively. The number of polyps in each case identified by the proposed system was the same as the number of polyps detected by endoscopists through per-polyp analysis. The recall rates for the high-quality image frames and polyp image frames were 95.7% and 92%, respectively. The overall alert accuracy rate and the false-positive rate for the low-quality dynamic images obtained through per-frame analysis were 95.3% and 0.18%, respectively.

## 4. Discussion

Nearly 70% of colorectal adenocarcinomas develop from conventional adenomas, with the remaining 30% develop from sessile serrated polyps [28]. The progression from polyp to adenocarcinoma generally occurs over 5–10 years [29], meaning that the incidence and mortality rates of colorectal cancer can be reduced through appropriate screening strategies. Several studies have indicated that appropriately performed screening colonoscopy and post-polypectomy colonoscopy surveillance can substantially decrease the incidence and mortality of colorectal cancer [5,30,31]. However, this protective effect is likely to be considerably compromised by low colonoscopy quality, which could increase the risk of post-colonoscopy colorectal cancer (PCCRC) [32].

Colorectal lesion detection is markedly affected by the quality of the colonoscopic image obtained, which is evaluated in terms of colon cleanliness, the clarity of mucosal images, and the degree of bowel distension [33]. Colon cleanliness refers to the degree of bowel cleanliness required for the careful examination of the mucosa after fecal water and residue have been suctioned. Although colon cleanliness is evaluated as excellent, good, fair, or poor, the differences between each level are not governed by any standardized criteria [34]. An alternative method is to assign quantitative scores based on the individual cleanliness of each region of the colon (e.g., Boston Bowel Preparation Scale, Ottawa Bowel Preparation Scale); however, these scoring systems are rather complicated [35,36]. Poor colon cleanliness prolongs the procedure duration and may lead to the missed detection of colorectal cancer or colorectal polyps. In addition, the early follow-up colonoscopy requirement, of within one year, because of poor colon cleanliness increases the economic burden on patients, medical institutions, and society as a whole [37]. The score calculated after colonoscopy cannot improve a patient’s bowel cleanliness for a current examination. Therefore, we developed an alert system to provide real-time feedback regarding the bowel cleanliness of a patient to the colonoscopist so that irrigation can be employed to clean the bowel lumen and improve the quality of the colonoscopy. Nevertheless, further clinical trials are required to verify whether this system can improve the detection rate of colorectal adenomas.

In addition to poor bowel preparation, another factor that may affect the clarity of mucosal images is the rapid withdrawal of the colonoscope, which can result in blurred images. According to multiple studies, physicians whose colonoscope withdrawal duration during a normal inspection is 6 min or longer have a considerably higher ADR than physicians whose withdrawal duration is less than 6 min [38]. In addition, retrospective studies have confirmed that the longer the colonoscope withdrawal duration, the lower is the risk of PCCRC [39]. Blurred images may cause physicians to miss colorectal cancers or polyps; therefore, alerting colonoscopists to unclear mucosal images may remind them to withdraw the colonoscope more slowly or to move the colonoscope back and forth in order to observe the unclear regions, and thus improve the examination quality. One clinical trial indicated that the use of the ENDOANGEL AI system, which features a built-in monitoring function for withdrawal speed, increases the ADR by 8% [40]. Another cause of blurred images is the unsteady movement of the colonoscope. Automatic quality-control AI systems can help monitor the stability of colonoscopy movement and alert physicians when an image is blurred [41]. One clinical trial revealed that such systems can increase the ADR by up to 12.4%.

Optimal colon distension is a prerequisite for colonoscopists to examine every part of the mucosa in detail. Several studies have confirmed that physicians who can adequately distend the lumen have a lower miss rate for colorectal adenomas [34]. A study comparing conventional colonoscopy with virtual colonoscopy indicated that approximately 11% of the polyps found, of which nearly 4% were adenomas larger than 6 mm [42], were missed with conventional colonoscopy. Most of these missed adenomas were located in the proximal part of a fold or near the anus orifice. Therefore, optimal distension can improve the ADR, and the real-time alert system proposed in the present study could alert endoscopists to the need to distend the lumen when a region is insufficiently inflated; such distension could in turn reduce the risk of clinicians failing to spot lesions.

The results of our study revealed that the proposed system could effectively alert endoscopists to low-quality images or poor colon preparation, prompting them to focus the image, clean the bowel, or inflate the lumen for detailed examination. This system reduces the likelihood of clinicians failing to spot colon polyps. The main limitation of this study was that only retrospectively recorded videos from a single medical center were used. Therefore, a large-scale prospective multicenter clinical trial is needed to validate the efficacy of the proposed system in increasing the colon polyp detection rate.

## 5. Conclusions

This study proposed an intraprocedure AI alert system for colonoscopy examination. Using feature extraction and classification alongside a CNN model, this system can identify blurred images, instances of inadequate bowel cleansing, and instances of insufficient air insufflation during colonoscopies. The system then alerts the clinician to the need to correct or pay greater attention to specific elements during examination in order to reduce the loss of crucial information and improve the reliability of the examination. The main novel feature of our study was the detection of low-quality images and foreign bodies in intestinal lumen to alert endoscopists and, thus, achieve higher-quality colonoscopy examination. Our experimental results indicated that blurred image and foreign body detection can prevent misjudgments and yield accurate polyp detection. In addition, our dynamic image sampling method indicated that the use of just three or four images from each second of footage for detection can yield accurate results; this means that our method is computationally lightweight.

Several polyp detection and classification systems can reduce the false-positive rate by enlarging training datasets. However, these systems still encounter challenges in the presence of colon folds, fecal matter, or water during examination. Our experimental results indicated that blurred image and foreign body detection can prevent misjudgments and be used to accurately detect polyps. Furthermore, thanks to our dynamic image sampling method, the use of just three or four images from each second of footage for detection can reduce the information processing load and thus lower the hardware requirements for image processing.

## Figures and Tables

**Table 1:** Training and validation image dataset.

Image Type		Image#
Blurred image		2500
Folds/fecal matter and water		1250
Good quality image	Polyp	1250
	Normal	1250
Total		6250

**Table 2:** Dynamic image test dataset.

Image Type		Image#
Blurred image		8716
Folds/fecal matter and water		1967
Good quality image	Polyp	50
	Normal	399
Total		11132

**Table 3:** Number of polyps.

Case#	Polyp#
1	2
2	1
3	1
4	1
5	1
6	1

**Table 4:** Polyp-detection training dataset.

Dataset	Image#	Size
CVC-ClinicDB-training	612	384 × 288
PolypsSet-training	500	640 × 480
Total	1112	

**Table 5:** CNN model of alert system.

Layers	Filters (N)	Size/Stride	Output (W × H)
Image Input			480 × 640
Convolution	16	(3 × 3)	480 × 640
Batch Normalization			480 × 640
ReLu			480 × 640
Max pooling		2 × 2/2	240 × 320
Convolution	16	(3 × 3)	240 × 320
Batch Normalization			240 × 320
ReLu			240 × 320
Max Pooling		2 × 2/2	480 × 640
Convolution	32	(3 × 3)	480 × 640
Batch Normalization			480 × 640
ReLu			480 × 640
Max Pooling		2 × 2/2	120 × 160
Convolution	32	(3 × 3)	120 × 160
Batch Normalization			120 × 160
ReLu			120 × 160
Max Pooling		2 × 2/2	60 × 80
Convolution	32	(3 × 3)	60 × 80
Batch Normalization			60 × 80
ReLu			60 × 80
Max Pooling		2 × 2/2	30 × 40
Convolution	32	(3 × 3)	30 × 40
Batch Normalization			30 × 40
ReLu			30 × 40
Max Pooling		2 × 2/2	15 × 20
Convolution	32	(3 × 3)	15 × 20
Batch Normalization			15 × 20
ReLu			15 × 20
Max Pooling		2 × 2/2	7 × 10
Fully Connected			7 × 10
Softmax			7 × 10
Classification Output			7 × 10

**Table 6:** CNN model of polyp detection.

Layers	Filters (N)	Size/Stride	Output (W × H)
Image Input			128 × 128
Convolution	32	(3 × 3 + 3 × 1 + 1 × 3)	128 × 128
Batch Normalization			128 × 128
ReLu			128 × 128
Max Pooling		2 × 2/2	64 × 64
Convolution	64	(3 × 3 + 3 × 1 + 1 × 3)	64 × 64
Batch Normalization			64 × 64
ReLu			64 × 64
Max Pooling		2 × 2/2	32 × 32
Convolution	128	(3 × 3 + 3 × 1 + 1 × 3)	32 × 32
Batch Normalization			32 × 32
ReLu			32 × 32
Max Pooling		2 × 2/2	16 × 16
Convolution	256	(3 × 3 + 3 × 1 + 1 × 3)	16 × 16
Batch Normalization			16 × 16
ReLu			16 × 16
Max Pooling		2 × 2/2	8 × 8
Convolution	256	(3 × 3 + 3 × 1 + 1 × 3)	8 × 8
Batch Normalization			8 × 8
ReLu			8 × 8
Convolution	256	(3 × 3 + 3 × 1 + 1 × 3)	8 × 8
Batch Normalization			8 × 8
ReLu			8 × 8
Convolution	256	(3 × 3 + 3 × 1 + 1 × 3)	8 × 8
Batch Normalization			8 × 8
ReLu			8 × 8
Convolution	24	1 × 1/1	8 × 8
Transform			8 × 8
Output			

**Table 7:** Training and validation datasets for blurred image detection and classification.

Image Type		Training Set#	Validation Set #	Total#
Blurred image		2000	500	2500
Good quality image	Polyp	1000	250	1250
	Normal	1000	250	1250
Subtotal		4000	1000	5000

**Table 8:** Training and validation datasets for foreign body detection and classification.

Image Type		Training Set#	Validation Set #	Total#
Folds/fecal matter and water image		1000	250	1250
Good quality image	Polyp	500	125	625
	Normal	500	125	625
Subtotal		2000	500	2500

**Table 9:** Validation results for blurred image detection.

	Blurred Image (Predicted)	Good Quality Image (Predicted)
Blurred image (Actual)	468 (TP)	32 (FN)
Good quality image (Actual)	6 (FP)	494 (TN)

**Table 10:** Validation results for detection of colon folds and fecal matter or water.

	Folds/Fecal Matter and Water Image (Predicted)	Good Quality Image (Predicted)
Folds/fecal matter and water image (Actual)	237 (TP)	13 (FN)
Good quality image (Actual)	6 (FP)	244 (TN)

**Table 11:** Performance index.

Accuracy (Acc)	$\mathrm{Acc}=\frac{\mathrm{TP}+\mathrm{TN}}{\mathrm{TP}+\mathrm{FP}+\mathrm{TN}+\mathrm{FN}}$	F1-measure (F1)	F1=2×Prec×RecPrec+Rec
Precision (Prec)	$\mathrm{Pr}\mathrm{ec}=\frac{\mathrm{TP}}{\mathrm{TP}+\mathrm{FP}}$	F2-measure (F2)	F2=5×Prec×Rec4×Prec+Rec
Recall (Rec)	Rec=TPTP+FN		

**Table 12:** Performance index for blurred image and foreign body detection.

	Acc%	Prec%	Rec%	F1%	F2%
Blurred image detection	96.2	98.8	93.6	96.1	94.6
Foreign body detection	96.2	97.5	94.8	96.1	95.3

**Table 13:** Total number of polyps.

Case#	Actual Polyp#	Predicted Polyp#
1	2	2
2	1	1
3	1	1
4	1	1
5	1	1
6	1	1
Total polyp	7	7

**Table 14:** Recall and false alarm rate for detection of image quality and polyp by per-frame analysis.

	Good Quality Image	Polyp Image	Total
Image#	399	50	449
Predicted#	382	46	428
Recall (%)	95.7	92	95.3
False alarm rate	21/11132 = 0.0018 = 0.18%		
